# Cross-cultural adaptation and psychometric validation of the GeKo^KIDS^ scale among Portuguese school-aged children

**DOI:** 10.1186/s12889-026-28771-6

**Published:** 2026-08-04

**Authors:** Maria João Batalha, Pedro Jesus, Anabela Graça, Ana Rita Pedro, Sara Simões Dias

**Affiliations:** 1 ciTechCare – Center for Innovative Care and Health Technology, Polytechnic of Leiria, Leiria, Portugal; 2 ENSP – Escola Nacional de Saúde Pública – Universidade NOVA de Lisboa, Lisboa, Portugal; 3 Câmara Municipal de Leiria, Leiria, Portugal; 4 School of Health Sciences, Polytechnic of Leiria, Leiria, Portugal; 5 CHRC – Comprehensive Health Research Center – Universidade NOVA de Lisboa, Lisboa, Portugal

**Keywords:** Health literacy, Cross-cultural adaptation, Psychometric validation, Test–retest reliability, Children

## Abstract

**Background:**

Promoting health literacy in childhood is essential, as it lays the foundation for lifelong healthy behaviors and supports informed decision-making. This study aimed to translate, cross-culturally adapt, and psychometrically validate the GeKo^KIDS^ scale for assessing health literacy in portuguese children, addressing the lack of validated instruments for this age group.

**Methods:**

The study was conducted at a school in Leiria, Portugal (October–December 2024), with a convenience sample of children aged 10–12 years. The cross-cultural adaptation followed standard procedures including translation–back translation, cognitive debriefing, and content validation by an expert panel. Psychometric evaluation included 83 participants, assessing internal consistency via Cronbach’s alpha and corrected item–total correlations. Factorial structure was examined using confirmatory factor analysis (CFA), with χ²/df, CFI, TLI, SRMR and RMSEA as fit indices. Test–retest reliability was assessed using the intraclass correlation coefficient (ICC (3,1)). Convergent validity was evaluated through correlations with perceived parental health guidance and motivation for health learning.

**Results:**

The adapted LiSa^KIDS^ retained the original five-domain structure comprising 16 items. Psychometric analyses were conducted on the 14 Likert-type items, resulting in a final 13-item psychometric model after the exclusion of one item. This model demonstrated acceptable-to-good internal consistency (Cronbach’s α = 0.807). CFA provided partial support for the proposed four-factor structure (χ²/df = 1.54; CFI = 0.94; TLI = 0.92; SRMR = 0.102; RMSEA = 0.081). Test–retest reliability indicated moderate-to-good temporal stability (ICC (3,1) = 0.724; 95% CI: 0.570–0.829; *p* < 0.001). Positive correlations were observed with parental health guidance (ρ = 0.417; *p* < 0.001) and motivation for health learning (ρ = 0.269; *p* = 0.015).

**Conclusions:**

LiSa^KIDS^ is a concise, reliable, and valid instrument for assessing health literacy in portuguese children. The scale demonstrated internal consistency, factorial validity, moderate to good temporal stability, and convergent validity, characteristics that support its use in research and public health initiatives aimed at promoting pediatric health literacy.

**Supplementary Information:**

The online version contains supplementary material available at https://doi.org/10.1186/s12889-026-28771-6.

## Background

Health literacy (HL) has become increasingly relevant, defined as a set of cognitive and social skills that enable individuals to access, comprehend, and apply information to promote and maintain health [1–3]. Developing HL in childhood is crucial for children’s physical, emotional, and cognitive growth, shaping their health-related behaviors [4]. HL equips young people to navigate information environments, critically evaluate messages, and make informed decisions, thereby fostering healthy behaviors and reducing preventable risks [5–7]. In Europe, the European Health Literacy Survey (HLS-EU) revealed that 47.6% of adults have limited health literacy (inadequate or problematic), with this figure rising to 61% in Portugal [8]. Most research in this area has focused on adults, and comparatively, the scientific literature on HL among children and adolescents remains scarce. This contrasts with the recognized importance of childhood and adolescence as critical periods for developing health skills^,^ acquiring health-related knowledge, and establishing healthy behaviors and practices [9–11]. The Health Behavior in School-Aged Children study was the first to provide comparative data on HL levels among 15-year-old adolescents, involving 10 countries that applied the Health Literacy for School-Aged Children scale. The study reported that 13.3% of respondents had low HL, 67.2% had moderate HL, and 19.5% had high HL. The results revealed variability in HL levels among adolescents across the 10 countries studied, with Macedonia and Finland exhibiting the highest proportion of youth with high HL and the lowest proportion with low HL, while Czechia showed the highest prevalence of low HL [12]. To date, and to the best of our knowledge, few studies have investigated HL in younger children, highlighting a significant gap in the literature. A systematic review conducted by Guo et al. identified 29 HL instruments for children and adolescents aged 6 to 24 years. The target populations of these instruments were classified into three subgroups: children aged 7 to 12 years (*n* = 5), adolescents aged 13 to 17 years (*n* = 20), and young adults aged 18 to 25 years (*n* = 4). Most of these instruments predominantly assess functional HL, focusing on basic reading and writing skills, with limited attention to the interactive and critical dimensions of HL. Among the five instruments targeting children up to 12 years old, the Gesundheitskompetenz bei Kindern in der Schule (GeKo^KIDS^) Scale stands out as a German self-report tool designed to assess both functional and interactive HL in children and adolescents aged 9 to 13 years. Based on Nutbeam’s health promotion outcome model, the scale comprises 17 items distributed across five key dimensions: knowledge, attitudes, communication, health-related behavior, and self-efficacy [3, 13].

## Methods

### Aim, design and setting of the study

This study aimed to culturally adapt the GeKo^KIDS^ instrument into Portuguese and to evaluate its psychometric properties in a sample of Portuguese youth aged 10–12 years. A two-phase methodological design was employed: Phase 1 comprised cross-cultural adaptation of the original instrument; and Phase 2 involved psychometric validation. The study was conducted in a single school in Portugal, involving students from the 5th and 6th grades.

### Characterization of the original instrument

The GeKo^KIDS^ was developed to assess core domains of HL in youth, based Nutbeam’s health promotion outcome model. Its five areas reflect key competencies: Knowledge assesses basic understanding of health information; Communication captures interactive skills for expressing concerns and engaging with others; Attitude reflects motivational factors influencing how health information is valued; Behavior indicates the ability to apply health knowledge in daily actions; and Self-efficacy represents confidence and autonomy in making informed health decisions [14]. The instrument consists of 17 items distributed across these five areas. Response scoring varies by item: some items range from 0 to 1, 0 to 2, 0 to 4 and 1 to 4.

### Participant recruitment and data collection

A convenience sample of 111 youths was recruited across two phases of the study: Phase 1, cross-cultural adaptation (*n* = 28), and Phase 2, psychometric validation (*n* = 83). Only students with informed consent from their legal guardians were included, and all data were collected anonymously and confidentially. Data collection took place between October and December 2024. The study followed institutional ethical guidelines and was approved by the Ethics Committee of the NOVA National School of Public Health (reference no. 28/2024).

### Inclusion/exclusion criteria for participants

The inclusion criteria for participants in this study consisted of youth aged between 10 and 12 years (5th and 6th grades) who demonstrated proficiency in understanding spoken and/or written Portuguese. Children with special educational needs that impeded their ability to complete the questionnaire independently were excluded.

### Cross-cultural adaptation

#### Translation and backtranslation

The first step in the cross-cultural adaptation of the GeKo^KIDS^ tool was to obtain authorization from the researchers who developed the instrument. Although the scale was originally developed for the German population, it is available in both German and English, with the English version available in the publication by Schmidt et al. [15–17]. After obtaining authorization, the translation–backtranslation methodology proposed by Hill and Hill was followed [18]. The initial version in English was independently translated by two translators, followed by a comparison of both proposals to create a single version. A backtranslation was then performed by an independent translator. The original and back translated versions were compared by a group of experts who assessed the content identity of the items and made the necessary corrections.

#### Pre-test and content validity

After the translation process, a pre-test was conducted by administering the translated version to 28 adolescents in the form of semi-structured interviews. Each participant first completed the scale derived from the translation. Subsequently, the participants were interviewed using an interview guide and an audio recorder to assess whether the items of the scale were correctly understood. This guide included the study objectives, key questions, and additional questions to be used only if the participant did not elaborate on the proposed topic. Through these interviews, it was possible to determine whether the adolescents believed there were items that needed improvement for better interpretation of the questions. The content of the interviews was transcribed and subsequently subjected to content analysis, which included pre-analysis of the transcripts, exhaustive exploration of the material, and grouping of the data into categories and subcategories. The analysis was carried out independently by three researchers, and all relevant information was subsequently organized in a table of categories and subcategories.

Simultaneously with the pre-test, a panel of ten experts in health and/or education was invited via email to evaluate the instrument’s items (title, instructions, and questions) regarding relevance, appropriateness, and clarity, using a scale from 1 (worst) to 5 (better). This request was accompanied by a detailed explanation of the study’s objectives. The Content Validity Index (I-CVI) was calculated for each item of the scale, as well as the Average Content Validity Index of the Scale (S-CVI/Ave). The I-CVI was computed by dividing the number of responses rated 4 and 5 by the total number of responses for each item. The S-CVI/Ave was obtained by summing the I-CVI values and dividing by the number of items. Items with an I-CVI < 0.83 were considered for revision based on expert feedback. For the review and refinement of the questions, both expert contributions and data collected from the student interviews were considered. The translated and culturally adapted version of the scale has been designated *Escala da Literacia em Saúde em crianças e adolescentes* (LiSa^KIDS^).

### Psychometric validation

Psychometric validation of the LiSa^KIDS^ scale was conducted in accordance with the COSMIN checklist [19]. The Knowledge domain was excluded exclusively from the psychometric analyses because its three items are multiple-choice questions with a single correct response, whereas the remaining domains comprise Likert-type items designed to assess latent constructs. Consequently, only the Likert-type component was included in the reliability and structural validity analyses. The Knowledge domain was, however, translated, culturally adapted, and retained in the final Portuguese version of the instrument. Data quality was assessed by mean, median and number of missing data. LiSa^KIDS^ reliability was evaluated through internal consistency, employing Cronbach’s alpha (α), the mean inter-item correlations, and the corrected item-total correlation. An α value exceeding 0.700 is deemed acceptable [20, 21]. The mean inter-item correlations should fall within the range of 0.150 to 0.500, ensuring that the items collectively measure the same construct while minimizing redundancy [22]. Furthermore, each item should exhibit a corrected item-total correlation with the overall construct greater than 0.300 [23]. Additionally, test–retest reliability was assessed in a subsample of 55 participants who completed the questionnaire a second time after a three-week interval, using the Intraclass Correlation Coefficient (ICC [3,1], two-way mixed-effects model, consistency). ICC values were interpreted as < 0.50 indicating poor reliability, 0.500–0.750 moderate, 0.750–0.900 good, and > 0.900 excellent [24]. To validate the internal structure of the questionnaire, a confirmatory factor analysis (CFA) was conducted to assess whether the observed data fit the theoretical model of the subscales proposed in the original German version. For the CFA, the Weighted Least Squares Mean and Variance Adjusted (WLSMV) estimator was used. Model fit was evaluated using the chi-square statistic (χ²), degrees of freedom (df), the chi-square to degrees of freedom ratio (χ²/df), the Comparative Fit Index (CFI), the Tucker–Lewis Index (TLI), the Standardized Root Mean Square Residual (SRMR), and the Root Mean Square Error of Approximation (RMSEA) with its 95% confidence interval. Model fit was evaluated according to the following criteria: χ²/df ≤ 2.0, CFI and TLI ≥ 0.90, SRMR ≤ 0.08, and RMSEA ≤ 0.08 [25]. Factor loadings below 0.300 are generally considered unacceptable, suggesting low relevance to the latent factor [26]. To assess convergent validity, bivariate correlation analyses (using Spearman’s rho) were conducted between the overall scale score and two theoretically related variables: perceived parental health orientation and motivation to acquire new health-related knowledge. Perceived parental health orientation was measured by averaging two items: *It’s important to my parents that I exercise regularly* and *My parents make sure that I have a healthy diet*. Motivation was measured with the statement: *I like to learn new subjects about health*. Responses were provided on a five-point Likert scale, ranging from 1 = “strongly disagree” to 5 = “strongly agree”.

### Statistical analysis software

The interview audio recordings were transcribed using the transcription feature in Microsoft Word. Content analysis was performed using NVivo version 1.6.1 (1137). For the CFA, R software version 4.4.2 was used. All other analyses were performed using the Statistical Package for the Social Sciences (SPSS) version 28.

## Results

### Cross-cultural adaptation

#### Pre-test and content validity

From the analysis of the content obtained in the interviews, three categories and six subcategories emerged (supplementary material). The category *Comprehension of the questionnaire content* includes the following subcategories: (1) *Vocabulary and language that are difficult to understand*, which identified terms such as *oral vaccine*, *administered*, *nutrition*, *incisors*, *teeth erupting*, *trouble* and *moderately* as difficult to understand; and (2) *Interpretation of the questions*, which pointed out formulation problems in questions 3 (*Which type of foods should be eaten or drunken most often during the day?*) and 15 (*I can solve most problems if I invest the necessary effort*). The *Self-Assessment of comprehension* category includes the following subcategories: (3) *Perception of the ease or difficulty of the questionnaire in general*, which revealed that most children considered the questionnaire easy to answer; and (4) *Most difficult items*, which identified difficulties related to the vocabulary of the answer options for question 1 (such as *incisors*, *teeth coming in* or *baby teeth*) and question 2 (*oral vaccine*, *injections in the arm* or *oral vaccine and injections in the arm*), as well as in the interpretation of questions 15 (*I can solve most problems if I invest the necessary effort*) and 16 (*If I am in trouble*,* I can usually think of a solution*). Finally, the category *Feedback on the questionnaire* includes the following subcategories: (5) *General opinions*, in which children expressed surprise regarding the question about tobacco consumption; and (6) *Proposals for modification*, where the suggestion was made to remove the *always* option from questions 4, 5 and 6.

The same scale was sent to the group of 10 experts, obtaining 8 responses. The S-CVI/Ave of the scale was 0.98, 0.88, and 0.84 for relevance, adequacy, and clarity, respectively (Table 1). The modifications that led to the final version of the scale were based particularly on suggestions regarding items with an I-CVI below 0.83, as well as on the most pertinent comments provided by the students. As part of the content validation process, the expert panel also recommended standardizing the response format to a five-point Likert scale. The original instrument included a combination of dichotomous, four-point Likert, and five-point Likert response formats. According to the experts, a uniform response format would improve the clarity, consistency, and usability of the questionnaire while reducing respondents’ cognitive burden. They also considered that the dichotomous response options did not adequately capture the full range of behaviours and experiences in the target population. Consequently, where appropriate, the response formats were standardized to a five-point Likert scale while preserving the conceptual intent of the original items. The response options of the first domain (Knowledge) were maintained in their original multiple-choice format because these items assess factual knowledge and therefore required a different response structure. Item 1 showed an I-CVI of 0.75 for relevance and 0.63 for clarity. Some experts considered it necessary to revise the question to avoid suggesting that certain teeth require more hygiene care than others, and also to simplify the response options by including examples. This aligns with the comments provide by the students in subcategory (1) *Vocabulary and language that are difficult to understand*. Item 2 also showed a clarity I-CVI below 0.83, with recurring issues identified regarding the vocabulary used. For this reason, the question was revised, along with the response options, with experts suggesting the use of the terms *injections* and *orally*. Item 3 raised concerns both among the expert panel and during the interviews, as it was unclear whether respondents were expected to select only one answer option. The I-CVI for clarity was 0.38, prompting a revision of the question. In items 4 and 5, vocabulary issues were identified, specifically with the term *nutrition* which was replaced by *diet*. Additionally, the children’s suggestion to replace *always* with *very often* was incorporated. This change was also implemented in item 6. Regarding items 7, 8, 10, and 12, although no content-related issues were identified, their response formats were standardized in accordance with the recommendation described above. According to the experts, the response options for item 9 could be improved; this was also taken into consideration and subsequently modified. Item 11 was identified as problematic during both the interviews and the expert panel assessment. In both instances, the question was deemed overly direct and potentially intimidating, leading to its complete reformulation. Although item 13 did not present an I-CVI below 0.83, it was also reviewed based on expert feedback, as the response options were considered limiting. Item 14 received a clarity I-CVI of 0.63. Experts indicated that both the question and the response options required revision. Feedback from the interviews aligned with the experts’ assessment, and as a result, the item was completely reformulated. Finally, although experts considered items 15, 16, and 17 to be relevant, clear, and appropriate, children demonstrated difficulties in interpreting the questions. As a result, the items were reformulated. The response formats were also standardized to the five-point Likert scale. These modifications resulted in the final Portuguese version of LiSa^KIDS^ questionnaire. The revised response format and corresponding scoring procedure are described in the following section. All changes made are shown in supplementary material.

**Table 1:** Content Validity Index (I-CVI) and S-CVI/Ave for the item relevance, adequacy, and clarity

Evaluated contents	Relevance	Adequacy	Clarity
	I-CVI	I-CVI	I-CVI
Title	1	0,88	0,75
Instructions	1	1	1
1. What teeth do you have to take care for in particular when tooth brushing?	0,88	0,75	0,63
2. How can vaccination be administered?	1	0,88	0,63
3. Which type of foods should be eaten or drunken most often during the day?	1	0,75	0,38
4. Did you talk already with your parents about healthy nutrition?	1	0,75	0,75
5. Did you talk already with your friends about healthy nutrition?	1	0,75	0,75
6. Did you talk already with your parents what’s good for your teeth?	1	1	1
7. Health is very important to me.	1	1	1
8. Healthy teeth are very important to me.	1	1	1
9. I like games in which I have to run a lot.	0,86	0,75	1
10. It is important to me to eat fruit and vegetables.	1	1	1
11. Do you smoke?	0,88	0,5	0,88
12. How often do you brush your teeth in the morning?	1	1	1
13. How often do you eat fruits a day?	1	0,88	0,88
14. How many hours per week do you exercise in a sports club?	1	0,88	0,63
15. I can solve most problems if I invest the necessary effort.	1	1	1
16. If I am in trouble, I can usually think of a solution.	1	1	1
17. I can usually handle any situation or obstacle I am faced with.	1	1	1
S-IVC/Ave	0,98	0,88	0,84

#### Pretest questionnaire

The translated and cross-culturally adapted version of the scale, LiSa^KIDS^, retained the original 17 items distributed across five domains: (1) Knowledge, (2) Communication, (3) Attitude, (4) Behavior, and (5) Self-efficacy. Following the content validation process, the response options of the Knowledge domain were maintained in their original multiple-choice format, while the response formats of the remaining domains were standardized to a five-point Likert scale. Accordingly, each Likert-scale item is scored from 1 to 5, whereas the multiple-choice items are scored as 1 for incorrect answers and 5 for correct answers. Percentage score for each subscale and the overall scale are calculated as follows: Percentage Score = [(subscale or scale score - minimum possible score on the subscale or scale) / (maximum possible score on the subscale or scale - minimum possible score on the subscale or scale)] × 100. Each response item on a Likert scale is scored from 1 to 5. The percentage score is further categorized into four levels: inadequate HL level for scores ≤ 25%, problematic HL level for scores between 25% and 50%, adequate HL level for scores between 50% and 75% and excellent HL level for scores > 75%.

### Psychometric validation

#### Participants

The sample comprised 83 students, including 28 (33.7%) from 5th grade and 55 (66.3%) from 6th grade, with slightly more boys (54.9%, *n* = 45) than girls (45.1%, *n* = 37). Most mothers (96.2%) and fathers (87.3%) had completed higher education. These results are shown in Table 2.

**Table 2:** Participant characteristics

		*n *(%)
Grade	Grade 5th	28 (33.7%)
	Grade 6th	55 (66.3%)
	Total	83 (100%)
Sex	Male	45 (54.9%)
	Female	37 (45.1%)
	Total	82 (100%)
Mothers’ education level	Basic Education	0 (0%)
	Secondary Education	3 (3.8%)
	Higher Education	75 (96.2%)
	Total	78 (100%)
Fathers’ education levels	Basic Education	1 (1.3%)
	Secondary Education	9 (11.4%)
	Higher Education	69 (87.3%)
	Total	79 (100%)

### Data quality

Most items showed negatively skewed distributions, indicating a tendency toward higher response categories. Items 6 and 2 were slightly skewed to the right, while Item 3 showed pronounced left skewness. Several items exhibited high kurtosis values, suggesting peaked response distributions. The response rate was high, with no missing data for most items and only one missing response observed in Item 2. These results are detailed in the supplementary material.

### Reliability and construct validity

**Fig. 1:**
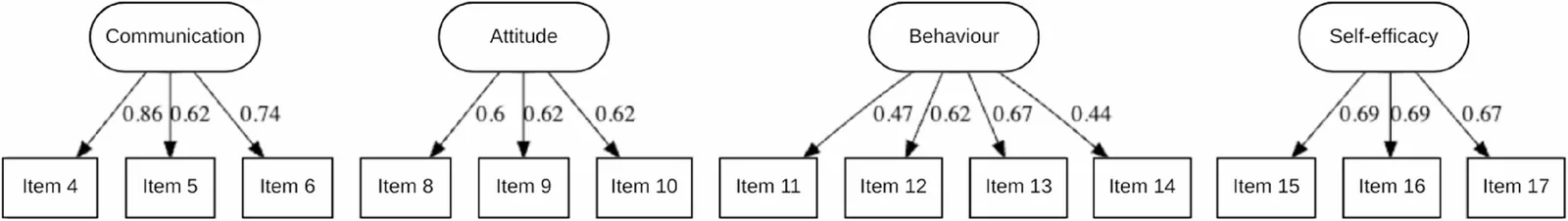
Four-factor measurement model of the 13-item LiSa^KIDS^ scale obtained through confirmatory factor analysis

Cronbach’s α coefficients for the 14-item LiSa^KIDS^ scale and its subscales are presented in Table 3. The overall scale demonstrated good internal consistency with a Cronbach’s α of 0.804. Subscale reliability coefficients ranged from 0.453 to 0.733. The average inter-item correlation was 0.23, falling within the theoretically acceptable range of 0.15 to 0.50. The corrected item-total correlations ranged from 0,174 (item 7) to 0.619 (item 4), with items 7 and 11 showing corrected item-total correlations below 0.30. The mean corrected item–total correlation for the 14-item LiSa^KIDS^ was 0.425, indicating an overall satisfactory relationship between the individual items and the total scale score. Regarding the analysis of Cronbach’s α if the item were removed, it was found that excluding items 7 and 11 would lead to an increase in the Cronbach’s value. These results are presented in Table 4. CFA indicated that all items exhibited factor loadings greater than 0.300, confirming their suitability for the instrument’s factorial structure (supplementary material). As shown in Table 5, CFA of the proposed 14-item four-factor model indicated an acceptable but not entirely satisfactory model fit (χ² = 104.8, df = 71, χ²/df = 1.48, CFI = 0.933, TLI = 0.915, SRMR = 0.173, RMSEA = 0.080, 90% CI: 0.042–0.106). Although χ²/df, CFI, TLI, and RMSEA indicated acceptable fit, the SRMR exceeded the recommended cutoff, suggesting that the proposed measurement model should be interpreted with caution. Considering these results, items 7 and 11 were re-evaluated as they met the following criteria: a higher Cronbach’s α after removal and corrected item-total correlations below 0.300. It was decided to retain Item 11, as it was deemed theoretically relevant. In contrast, Item 7, was removed due to the likelihood of social desirability bias, which could lead to responses that do not accurately reflect the respondent’s true feelings. After removing item 7, Cronbach’s α for the remaining 13 items was 0.807, exceeding the value observed in the initial 14-item version. Item 11 continued to show a corrected item-total correlation below 0.300, and its removal would result in an increase in the α value. In the CFA, all items continued to exhibit factor loadings above 0.300 (Fig. 1), supporting their retention in the four-factor model. Compared with the 14-item version, the 13-item model showed a slight improvement in model fit, as reflected by higher CFI (0.940) and TLI (0.920) values and a lower χ² (χ²/df = 1.54). The RMSEA (0.081) remained at the threshold of acceptable fit, whereas the SRMR (0.102) was reduced but still exceeded the recommended cutoff. Overall, these findings suggest a modest improvement in model fit, although the results should still be interpreted with caution. The ICC (3,1) was 0.724 (95% CI: 0.570–0.829; *p* < 0.001), indicating moderate-to-good test–retest reliability.

**Table 3:** Cronbach’s α coefficient for 14-item and 13-item LiSa^KIDS^ scale and its subscales

	Number of items	Cronbach’s α	Number of items	Cronbach’s α
LiSa^KIDS^	14	0.804	13	0.807
Communication	3	0.733	3	0.733
Attitude	4	0.488	3	0.471
Behaviour	4	0.453	4	0.453
Self-efficacy	3	0.606	3	0.606

**Table 4:** Corrected item-total correlations and Cronbach’s α if item deleted for 14- and 13-item LiSa^KIDS^ scales

	Corrected item-total correlation 14-item	Cronbach’s α if item was deleted 14-item	Corrected item-total correlation 13-item	Cronbach’s α if item was deleted 13-item
Item 4	0.619	0.776	0.615	0.779
Item 5	0.444	0.791	0.448	0.794
Item 6	0.524	0.783	0.529	0.786
Item 7	0.174	0.807	-	-
Item 8	0.372	0.798	0.368	0.802
Item 9	0.486	0.787	0.483	0.791
Item 10	0.397	0.796	0.386	0.800
Item 11	0.197	0.807	0.200	0.810
Item 12	0.445	0.791	0.440	0.794
Item 13	0.487	0.787	0.489	0.790
Item 14	0.302	0.803	0.301	0.806
Item 15	0.529	0.784	0.531	0.787
Item 16	0.523	0.784	0.520	0.787
Item 17	0.450	0.791	0.453	0.794

**Table 5:** Model fit results for the model of 14-item and 13-item LiSa^KIDS^ scale

Model	Χ²	gl	Χ²/gl	CFI	TLI	SRMR	RMSEA (IC90%)
14-item	104.8	71.0	1.48	0.933	0.915	0.173	0.08 (0.042–0.106)
13-item	91.0	59	1.54	0.940	0.920	0.102	0.081 (0.046–0.113)

### Convergent validity

A statistically significant positive correlation was found between perceived parental health orientation and the overall scale score (*ρ* = 0.417; *p* < 0.001), indicating a positive moderate correlation. Additionally, interest in learning new health topics was significantly positively correlated with the overall scale score (*ρ* = 0.269; *p* = 0.015), reflecting a weak but meaningful relationship between these variables.

### Percentage score of the participants

After removing item 7, the percentages obtained for each participant were calculated. The results of the LiSa^KIDS^ scale administration revealed that most students demonstrated satisfactory levels of HL. Overall, 53.7% of the participants were classified as having adequate HL, while 41.5% reached an excellent level; only 4.9% were categorized as having problematic HL, and none were classified as inadequate. In the Knowledge subscale, 45.1% of students achieved an adequate level and 20.7% an excellent level, although 34.1% were found to have problematic HL. The Communication subscale showed a broader distribution, with 47.0% at an adequate level, 33.7% problematic, and 12.0% inadequate. For the Attitude subscale, results were overwhelmingly positive: 83.1% of students scored at an excellent level, and none were classified as inadequate. Similarly, in the Behavior subscale, 74.7% reached an excellent level and 20.5% an adequate level. The Self-efficacy subscale revealed more variability, with 31.3% rated excellent, 37.3% adequate, 28.9% problematic, and 2.4% inadequate (Table 6). No statistically significant differences were observed between sexes in any of the subscales or in the overall score (*p* > 0.05 for all comparisons). No statistically significant differences were found between 5th and 6th grade students in the overall LiSa^KIDS^ score (*p* = 0.236) or across any of its subscales (*p* > 0.05 for all comparisons). Detailed results of these comparisons in the supplementary material.

**Table 6:** Score of the participants

		*n *(%)
LiSa^KIDS^ (Total)	Inadequate	0 (0.0%)
	Problematic	4 (4.9%)
	Adequate	44 (53.7%)
	Excellent	34 (41.5%)
	Total	82 (100.0%)
Knowledge	Inadequate	0 (0.0%)
	Problematic	28 (34.1%)
	Adequate	37 (45.1%)
	Excellent	17 (20.7%)
	Total	82 (100.0%)
Communication	Inadequate	10 (12.0%)
	Problematic	28 (33.7%)
	Adequate	39 (47.0%)
	Excellent	6 (7.2%)
	Total	83 (100.0%)
Attitude	Inadequate	0 (0.0%)
	Problematic	1 (1.2%)
	Adequate	13 (15.7%)
	Excellent	69 (83.1%)
	Total	83 (100.0%)
Behaviour	Inadequate	0 (0.0%)
	Problematic	4 (4.8%)
	Adequate	17 (20.5%)
	Excellent	62 (74.7%)
	Total	83 (100.0%)
Self-efficacy	Inadequate	2 (2.4%)
	Problematic	24 (28.9%)
	Adequate	31 (37.3%)
	Excellent	26 (31.3%)
	Total	83 (100.0%)

## Discussion

To our knowledge, this study is the first attempt to translate, adapt and validate a HL scale for Portuguese youth aged between 10 and 12 years. LiSa^KIDS^ scale presents 16 items, including 3 multiple-choice questions and 13 Likert-scale items, distributed across the five original subscales. The cross-cultural adaptation methodology incorporated the perspectives of both experts and children, and the qualitative analysis revealed difficulties in understanding certain terms (e.g., *incisors*, *nutrition*, *injections*, *moderately*, *oral vaccination*), highlighting the need to adjust the vocabulary to the cognitive and linguistic level of the target age group. This concern has already been noted in previous studies, which emphasize semantic clarity and conceptual, linguistic, and cultural equivalence as essential to ensuring the validity of health literacy instruments [27–30]. The expert evaluation revealed high indices of relevance, appropriateness, and clarity (S-CVI/Ave = 0.98, 0.88, and 0.84, respectively), supporting the adequacy of the instrument. The simultaneous incorporation of feedback from both participants and experts further strengthens the robustness of the adaptation, as it ensures the theoretical relevance of the items while also guaranteeing their comprehensibility in practice. In addition to the linguistic and cultural adaptations, the response format was modified to improve its comprehensibility and developmental appropriateness for Portuguese children aged 10–12 years. This decision was informed by the cross-cultural adaptation process and aimed to facilitate accurate interpretation of the items while preserving the original conceptual framework and HL domains. Although this modification enhanced the instrument’s suitability for the target population, it should be acknowledged that it may limit the direct comparability of scores with those obtained using the original German version.

Psychometric evaluation was conducted exclusively on the 14 Likert-type items comprising the Communication, Attitude, Behavior, and Self-efficacy domains. Accordingly, the psychometric findings reported in this study apply only to these four domains. The Knowledge domain was translated, culturally adapted, and retained in the final Portuguese version of the instrument but was not included in the psychometric evaluation because it comprises multiple-choice items with a distinct response format. LiSa^KIDS^ demonstrated good overall internal consistency (Cronbach’s α = 0.804), supporting its reliability as a measure of HL in children aged 10 to 12 years. Nonetheless, some subscales yielded Cronbach’s α coefficients below the conventional threshold of 0.700. These results should be interpreted with caution, as Cronbach’s α is sensitive to the number of items in a scale. It is well documented that subscales comprising a limited number of items, particularly those with three to five, often produce α values below 0.700, even when items are conceptually coherent and moderately correlated [31, 32]. Therefore, the lower coefficients observed in certain subscales of the LiSa^KIDS^ do not necessarily indicate inadequate internal consistency but rather reflect the statistical limitations of Cronbach’s α in scales with fewer items [33]. Items 7 and 11 showed corrected item-total correlations below the recommended threshold of 0.300, indicating weaker alignment with the overall construct. Although removing either item would have increased Cronbach’s α, different decisions were made regarding their retention. Item 11 was retained despite its corrected item–total correlation remaining below the recommended threshold (< 0.300) because of its theoretical relevance to the self-efficacy construct, given that tobacco use represents one of the greatest global public health threats, accounting for over 8 million deaths annually [34]. This concern is further underscored by data from the WHO Global Report 2000–2025, which indicate that at least 37 million adolescents aged 13 to 15 years (9.7%) used some form of tobacco worldwide, rising to 4 million (10.8%) in the WHO European Region [35]. Therefore, although the retention of Item 11 represents a psychometric limitation, its conceptual and public health relevance was considered to outweigh the modest statistical improvement associated with its exclusion. The decision to remove Item 7 was not based solely on statistical criteria. Although its exclusion resulted in only a slight increase in Cronbach’s α, the item consistently demonstrated weak psychometric performance, including a low corrected item-total correlation, and was considered particularly susceptible to social desirability bias. Therefore, the decision combined empirical evidence with theoretical considerations regarding the quality of measurement [36]. After removing item 7, Cronbach’s α increased slightly to 0.807. The CFA provided evidence supporting the construct validity of the Portuguese LiSa^KIDS^. All retained items showed adequate factor loadings, supporting preservation of the original four-factor structure. Although the fit indices generally supported the proposed model, the elevated SRMR values indicate that the overall model fit should be interpreted with caution. These findings suggest that the underlying factorial structure of the original instrument was largely preserved in the Portuguese version, supporting its suitability for assessing HL in Portuguese children. However, further studies are needed to establish cross-cultural measurement invariance before direct comparisons between language versions can be made [16]. The removal of Item 7 resulted in a modest improvement in model fit, reflected by slightly higher CFI and TLI values and a lower SRMR, although the SRMR remained above the recommended threshold. The comparison between the 14- and 13-item models was undertaken to evaluate the performance of the translated items in the Portuguese population and to determine whether any item required modification to ensure the psychometric adequacy of the adapted version. The significant positive correlations observed between the overall LiSa^KIDS^ score and both perceived parental health orientation (*ρ* = 0.417; *p* < 0.001) and interest in learning new health topics (ρ = 0.269; *p* = 0.015) provide evidence of the scale’s convergent validity. Children who perceive greater parental engagement in health-related behaviors or who express higher motivation to learn about health tend to achieve higher LiSa^KIDS^ scores, consistent with theoretical expectations [37–39].

In this study, most adolescents demonstrated adequate (53.7%) or excellent (41.5%) levels of HL, while only 4.9% were classified as having problematic levels and none as inadequate. Although these results cannot be directly compared, given that this was the first application of the LiSa^KIDS^ as an assessment tool, they can be cautiously contextualized in relation to existing evidence. A systematic review evaluating HL among students aged 6 to 18 years reported substantially higher proportions of low and moderate levels, in contrast with the more favorable outcomes observed in the present study [40]. Such discrepancies may reflect methodological variations, cultural and contextual differences, as well as the diversity of instruments employed. It is also noteworthy that the review encompassed a broader age range, which naturally influences the findings, as the literature indicates that older students generally demonstrate significantly higher health literacy compared to younger students [40, 41]. Additionally, the high educational attainment of parents in this sample may have contributed to the elevated HL levels observed, as parental education is widely recognized as a key social determinant of children’s HL [37]. Contrary to findings from previous studies, no statistically significant differences in HL scores were observed between boys and girls or between 5th- and 6th-grade students [37, 42]. However, these findings should be interpreted with caution, as the present study was designed primarily for the psychometric validation of the LiSa^KIDS^ rather than for subgroup comparisons. Consequently, the sample size may have been insufficient to detect small or moderate between-group differences, and the absence of statistically significant differences should not be interpreted as evidence of equivalence between groups. Future studies with larger samples specifically powered for subgroup analyses are warranted.

Notably, research on HL in younger age groups remains limited, underscoring the need for further studies to better characterize HL levels and enable meaningful comparisons across populations. The LiSa^KIDS^ scale has proven to be a concise and reliable instrument for assessing HL in Portuguese children aged 10 to 12 years. Its application can provide valuable insights to inform the development and implementation of public health initiatives aimed at enhancing literacy and promoting healthier behaviors among children.

One of the main strengths of this study lies in the rigorous methodology applied to the translation and cross-cultural adaptation of the scale, which combined a qualitative pretest with semi-structured interviews, ensuring item clarity and appropriateness from the children’s perspective, with an evaluation of relevance, adequacy, and clarity conducted by a multidisciplinary panel of experts. In addition, the use of robust psychometric analyses strengthens the credibility of the findings, supporting both the reliability and validity of the instrument. Notably, the LiSa^KIDS^ is grounded in a solid theoretical framework, assessing multiple domains of HL, namely knowledge, attitudes, communication, behaviors, and self-efficacy, which can be considered key health promotion outcomes according to Nutbeam’s model [16, 43]. Furthermore, the LiSa^KIDS^ is concise and quick to complete, making it a practical and user-friendly tool for assessing and monitoring HL levels in health promotion contexts. Nevertheless, certain limitations should be acknowledged. Despite the sample size being deemed adequate according to COSMIN checklist, the relatively small and sociodemographically skewed sample, notably the high proportion of parents with higher education, may have influenced the elevated health literacy levels observed [19]. In addition, although the CFA supported the proposed factorial structure, the limited sample size (*n* = 83) may have affected the stability of the fit indices, particularly in analyses of ordinal data estimated using WLSMV. Therefore, replication of the factorial structure in larger and more heterogeneous samples is warranted. Furthermore, the possibility of social desirability bias cannot be excluded, as children may have provided responses they perceived as socially acceptable rather than reflecting their true opinions or behaviours [36]. Convergent validity was examined using theoretically relevant variables developed for the purposes of this study, as no validated comparator instrument assessing related constructs was available for Portuguese children in this age group. Therefore, these findings should be considered preliminary and interpreted with appropriate caution. Moreover, modifications to the response format and the exclusion of one item following psychometric evaluation limit the direct comparability of scores between the Portuguese and the original German versions. Although the conceptual framework of the instrument was preserved, formal cross-cultural measurement invariance studies are required before score equivalence between language versions can be established. Finally, the psychometric evaluation was limited to the Communication, Attitude, Behavior, and Self-efficacy domains because the Knowledge domain comprises multiple-choice items with a distinct response format. Consequently, the measurement properties of the Knowledge domain were not examined in the present study and remain to be established using psychometric approaches appropriate for knowledge-based items.

## Conclusion

LiSa^KIDS^ is a concise, user-friendly instrument grounded in a solid theoretical framework. The present findings provide initial evidence supporting its reliability and validity for assessing multiple domains of HL in Portuguese youth aged 10–12 years. Its application may contribute to the assessment of youth’s HL and provide valuable information to inform health promotion strategies and support the development of healthier behaviours from an early age.

## Supplementary Information

Supplementary Material 1.

## Data Availability

The data supporting this study’s findings were obtained from the ATIVA+Saúde project and consist of anonymized information collected from youth in a school setting. Due to ethical considerations and consent restrictions, the dataset is not publicly available.

## Abbreviations

GeKo^KIDS^: Gesundheitskompetenz bei Kindern in der Schule
LiSaKIDS: Escala da Literacia em Saúde em crianças e adolescentes
CFA: Confirmatory factor analysis
ICC: Intraclass correlation coefficient
HL: Health literacy
HLS-EU: European Health Literacy Survey
I-CVI: Content Validity Index
S-CVI/Ave: Average Content Validity Index of the Scale
X²/df: Chi-square to degrees of freedom ratio
CFI: Comparative Fit Index
TLI: Tucker-Lewis Index
SRMR: Standardized Root Mean Square Residual
RMSEA: Root Mean Square Error of Approximation
