# Supplementary material for: Cross-cultural adaptation and psychometric validation of the GeKoKIDS scale among Portuguese school-aged children
Source: BMC Public Health. 2026 Aug 4;26:3142. doi: 10.1186/s12889-026-28771-6 (PMC13721346; doi:10.1186/s12889-026-28771-6)
Supplement: Supplementary file 1 [file 12889_2026_28771_MOESM1_ESM.docx]

Table1: Results of the content analysis of the interviews conducted during the pre-test

| Category | Subcategory | Interview transcripts | Conclusion |
| --- | --- | --- | --- |
| Comprehension of the questionnaire content | Vocabulary and language that are difficult to understand | “Oral vaccine? What do you mean by oral vaccine?”  “I don’t know what an oral vaccine is.”  “In the administered one, I didn’t really know what it was.”  “I don’t really know what oral vaccination is — whether it’s talking or injecting in the cheek.”  “I didn’t know what an oral vaccine was.”  “I didn’t quite understand what an oral vaccine was and the injection in the arm.”  “I couldn’t understand the vaccination in the arm and the oral one; I didn’t understand the oral one.” | Question 2  Oral vaccine  Administered |
|  |  | “Yes, nutrition is nutrients.” | Question 4 and 5  Nutrition |
|  |  | “I also didn’t know what ‘teeth erupting’ meant.”  “I don’t know what an incisor is.”  “I didn’t understand what an incisor was.”  “I didn’t understand the question about teeth that are erupting. What does erupting mean?”  “I found it a bit difficult because of the names of the teeth.”  “I thought the question was easy, but I didn’t know what an incisor was.”  “I found it easy, but I also didn’t know what incisors were or what teeth erupting meant.”  “The only thing I didn’t understand was ‘incisor’.”  “I found it a little confusing because I didn’t know the names of the teeth.”  “I didn’t understand the one about teeth, incisors.”  “I thought it was a bit strange because I didn’t know what incisors were and what teeth erupting meant.” | Question 1  Incisor  Teeth erupting |
|  |  | “I don’t know what ‘troubles’ are.”  “I sort of know what ‘troubles’ are.” | Question 16  Troubles |
|  |  | “I didn’t really understand what ‘moderately’ means.”  “I didn’t really understand this part here about ‘moderately true’.” | Question 7, 8, 15, 16 e 17  Moderately |
|  | Interpretation of the questions | “I didn’t understand the effort required for that, like for that.” | Question 15  Issues in question formulation. |
|  |  | “At first, I even chose both, but then I remembered I could only choose one.”  “Here I was unsure about this one because I didn’t know if it was water or fruits and vegetables.”  “I found it a bit difficult because I thought there were two correct answers… but then I realized it was water.” | Question 3  It was not clear that only one option should be selected. |
| Self-Assessment of comprehension | Perception of the ease or difficulty of the questionnaire in general | “So-so.”  “It was very easy.”  “It was easy.”  “It was easy, yes, it was easy.”  “It was easy, I thought it was easy, just normal.”  “Super easy.”  “Easy.”  “I think the questionnaire was easy, but there were a few questions that were difficult to answer.”  “I think they were all easy.”  “I think some questions were a little more difficult, but overall it was all easy.”  “Easy.” | Most children found the questionnaire easy to complete. |
|  | Most difficult items | “Maybe number 1 too, because of the incisors.”  “The question about teeth was difficult.”  “I found the question about teeth difficult.”  “The first questions? Which tooth should you brush best?”  “The one about teeth.”  “And that one about teeth.” | Question 1: Which teeth should you brush most carefully?  Difficulty: Vocabulary used in the response options. |
|  |  | “And I also found number 2 a little difficult.” | Question 2: How can vaccination be administered?  Difficulty: Vocabulary used in the response options. |
|  |  | “And number 15 too.”  “And I didn’t understand number 15 either.” | Question 15: I can solve most problems if I invest the necessary effort.  Difficulty: Difficulty in interpreting the question. |
|  |  | “Number 17, the last one.”  “I would say the last three are the most difficult to understand.” | Question 16: If I am in trouble, I can usually think of a solution.  Difficulty: Lack of understanding of the word troubles.  Question 17: I can usually handle any situation or obstacle I am faced with.  Difficulty: Difficulty in interpreting the question; possible lack of understanding of the word faced. |
| Feedback on the questionnaire | General opinions | “The question about smoking is really silly; our parents would have killed us.” |  |
|  | Proposals for modification | “Only the first one; I would remove the question.”  “I would change ‘faced’ to ‘a fight’.”  “Not always; I can’t, when I get home, always be talking about the same thing. That’s why I put ‘many times.’ I don’t understand the logic of having ‘always’ here. It doesn’t make sense.”  “I would only include the question about smoking for certain ages.”  “One question that could be added for older children: ‘Do you consume alcoholic beverages?’” | Remove the first question.  Reword question 17 and replace the word “faced.”  Remove the “always” option from questions 4, 5, and 6.  Restrict the question about smoking habits to older children.  Add a question about alcohol consumption. |

Table 2: Modifications made to the items of the Health Literacy Scale for children and adolescents after content validation

| Translated version | Final version | Changes resulting from | |
| --- | --- | --- | --- |
|  |  | Interviews | Experts |
| 1. What teeth do you have to take care for in particular when tooth brushing?  ▢ Incisors  ▢ Teeth that are erupting  ▢ Baby teeth | 1. Which teeth should you pay the most attention to when brushing?  ▢ Incisors (front teeth)  ▢ Teeth that are erupting  ▢ Baby teeth  ▢ I don’t know | ✖ | ✖ |
| 2. How can vaccination be administered?  ▢ Oral vaccine  ▢ Injections in the arm  ▢ Oral vaccine and injections in the arm | 2. What are the ways to receive a vaccine?  ▢ Oral  ▢ Injections  ▢ Oral and injections  ▢ I don’t know | ✖ | ✖ |
| 3. Which type of foods should be eaten or drunken most often during the day?  ▢ Sweets  ▢ Bread  ▢ Fruits and vegetables  ▢ Water  ▢ Lemonade | 3. Which food or drink should be consumed most often during the day?  ▢ Sweets  ▢ Bread  ▢ Fruits and vegetables  ▢ Water  ▢ Lemonade  ▢ I don’t know | ✖ | ✖ |
| 4. Did you talk already with your parents about healthy nutrition?  ▢ Never  ▢ Rarely  ▢ Sometimes  ▢ Often  ▢ Always | 4. Did you talk already with your parents about healthy diet?  ▢ Never  ▢ Rarely  ▢ Occasionally / Sometimes  ▢ Frequently  ▢ Very frequently | ✖ | ✖ |
| 5. Did you talk already with your friends about healthy nutrition?  ▢ Never  ▢ Rarely  ▢ Sometimes  ▢ Often  ▢ Always | 5. Did you talk already with your friends about healthy diet?  ▢ Never  ▢ Rarely  ▢ Occasionally / Sometimes  ▢ Frequently  ▢ Very frequently | ✖ | ✖ |
| 6. Did you talk already with your parents what’s good for your teeth?  ▢ Never  ▢ Rarely  ▢ Sometimes  ▢ Often  ▢ Always | 6. Did you talk already with your parents what’s good for your teeth?  ▢ Never  ▢ Rarely  ▢ Occasionally / Sometimes  ▢ Frequently  ▢ Very frequently | ✖ |  |
| 7. Health is very important to me.  ▢ Not true at all  ▢ Slightly true  ▢ Moderately true  ▢ Completely true | 7. Health is very important to me.  ▢ Strongly disagree  ▢ Disagree  ▢ Neither agree nor disagree  ▢ Agree  ▢ Strongly agree |  |  |
| 8. Healthy teeth are very important to me.  ▢ Not true at all  ▢ Slightly true  ▢ Moderately true  ▢ Completely true | 8. Healthy teeth are very important to me.  ▢ Strongly disagree  ▢ Disagree  ▢ Neither agree nor disagree  ▢ Agree  ▢ Strongly agree |  |  |
| 9. I like games in which I have to run a lot.  ▢ Yes  ▢ No | 9. I like games in which I have to run a lot.  ▢ Strongly disagree  ▢ Disagree  ▢ Neither agree nor disagree  ▢ Agree  ▢ Strongly agree |  | ✖ |
| 10. It is important to me to eat fruit and vegetables.  ▢ Yes  ▢ No | 10. It is important to me to eat fruit and vegetables.  ▢ Strongly disagree  ▢ Disagree  ▢ Neither agree nor disagree  ▢ Agree  ▢ Strongly agree |  |  |
| 11. Do you smoke?  ▢ Yes  ▢ No | 11. I think I might try smoking one day.  ▢ Strongly agree  ▢ Agree  ▢ Neither agree nor disagree  ▢ Disagree  ▢ Strongly disagree | ✖ | ✖ |
| 12. How often do you brush your teeth in the morning?  ▢ Not every day  ▢ Every day | 12. How often do you brush your teeth in the morning?  ▢ Never  ▢ Rarely  ▢ Occasionally / Sometimes  ▢ Often  ▢ Always |  |  |
| 13. How often do you eat fruits a day?  ▢ 3 times or less  ▢ More than 3 time | 13. I eat between 3 and 5 pieces of fruit per day.  ▢ Never  ▢ Rarely  ▢ Occasionally / Sometimes  ▢ Often  ▢ Always |  | ✖ |
| 14. How many hours per week do you exercise  in a sports club?  ▢ Less than 2 hours per week  ▢ 2 hours or more per week | 14. How many hours per week do you practice a sport outside of physical education?  ▢ I don’t practice  ▢ Up to 1 hour  ▢ Between 1 and 2 hours  ▢ Between 2 and 3 hours  ▢ More than 3 hours | ✖ | ✖ |
| 15. I can solve most problems if I invest the necessary effort.  ▢ Not true at all  ▢ Slightly true  ▢ Moderately true  ▢ Exactly true | 15. If I make an effort, I can solve most problems.  ▢ Strongly disagree  ▢ Disagree  ▢ Neither agree nor disagree  ▢ Agree  ▢ Strongly agree | ✖ |  |
| 16. If I am in trouble, I can usually think of  a solution.  ▢ Not true at all  ▢ Slightly true  ▢ Moderately true  ▢ Exactly true | 16. When I am upset or nervous, I can think of a solution.  ▢ Strongly disagree  ▢ Disagree  ▢ Neither agree nor disagree  ▢ Agree  ▢ Strongly agree | ✖ |  |
| 17. I can usually handle any situation or obstacle I am faced with.  ▢ Not true at all  ▢ Slightly true  ▢ Moderately true  ▢ Exactly true | 17. I can handle any problem that comes my way.  ▢ Strongly disagree  ▢ Disagree  ▢ Neither agree nor disagree  ▢ Agree  ▢ Strongly agree | ✖ |  |

Table 3: Data quality (descriptive statistics)

|  | LiSa^KIDS^ | Mean | Standard Deviation | Mode | Skewness | Kurtosis | Missing |
| --- | --- | --- | --- | --- | --- | --- | --- |
| Communication | Item 4 | 3.49 | .929 | 3^a^ | -.309 | .019 | 0 |
|  | Item 5 | 3.61 | 1.069 | 3 | -.890 | .574 | 0 |
|  | Item 6 | 2.39 | 1.080 | 4 | .482 | -.164 | 0 |
| Attitude | Item 7 | 4.96 | .188 | 5 | -5.062 | 24.210  13.585 | 0 |
|  | Item 8 | 4.81 | .480 | 5 | -3.211 |  | 0 |
|  | Item 9 | 4.14 | .926 | 5 | -1.146 | 1.555 | 0 |
|  | Item 10 | 4.66 | .590 | 5 | -1.941 | 4.501 | 0 |
| Behaviour | Item 11 | 4.75 | .730 | 5 | -3.229 | 10.856 | 0 |
|  | Item 12 | 4.53 | 1.052 | 5 | -2.435 | 5.031 | 0 |
|  | Item 13 | 3.89 | 1.012 | 4 | -.718 | -.198 | 0 |
|  | Item 14 | 4.42 | .989 | 4 | -1.791 | 2.653 | 0 |
| Self-efficacy | Item 15 | 4.28 | .888 | 5 | -1.545 | 3.068 | 0 |
|  | Item 16 | 3.35 | 1.098 | 4 | -.794 | -.107 | 0 |
|  | Item 17 | 3.33 | 1.116 | 4 | -.356 | -.472 | 0 |

^a^ There are multiple modes. The lowest value is reported.

Table 4: Factor loadings for the model of 14-item LiSa^KIDS^ scale

|  | LiSa^KIDS^ | Std. all |
| --- | --- | --- |
| Communication | Item 4 | 0.873 |
|  | Item 5 | 0.612 |
|  | Item 6 | 0.731 |
| Attitude | Item 7 | 0.787 |
|  | Item 8 | 0.640 |
|  | Item 9 | 0.633 |
|  | Item 10 | 0.728 |
| Behaviour | Item 11 | 0.471 |
|  | Item 12 | 0.631 |
|  | Item 13 | 0.667 |
|  | Item 14 | 0.424 |
| Self-efficacy | Item 15 | 0.682 |
|  | Item 16 | 0.692 |
|  | Item 17 | 0.672 |

Table 5: Comparison of LiSaKIDS Scores between male and female students for general scale and for each subscale

|  | Female | Male | p-value* |
| --- | --- | --- | --- |
|  | Median (Q1 – Q3) | Median (Q1 – Q3) | Bilateral |
| LiSa^KIDS^ | 71.87 (65.63 – 78.13) | 73.44 (61.72 – (82.42) | 0.732 |
| Knowledge  (Item 1 a item 3) | 66.67 (33.33 – 83.33) | 66.67 (33.33 – 66.67) | 0.902 |
| Communication (Item 4 a item 6) | 58.33 (45.83 – 66.67) | 58.33 (33.33 – 66.67) | 0.191 |
| Attitude  (Item 7 a item 10) | 91.67 (83.33 – 100.00) | 91.67 (83.33 – 100.00) | 0.915 |
| Behaviour  (Item 11 a item 14) | 87.50 (68.75 – 93.75) | 93.75 (81.25 – 100.00) | 0.069 |
| Self-efficacy  (Item 15 a item 17) | 66.67 (50.00 -83.33) | 66.67 (50.00 -83.33) | 0.910 |

*The Mann–Whitney U test was used due to the non-normal distribution of the data.

Table 6: Comparison of LiSaKIDS Scores between 5th and 6th students for general scale

|  | Grade 5th | Grade 6th | p-value |
| --- | --- | --- | --- |
|  | Mean ± SD | Mean ± SD | Bilateral |
| LiSa^KIDS^ | 74.22 ± 10.64 | 70.80 ± 13.04 | 0.236 |

Table 7: Comparison of LiSaKIDS Scores between 5th and 6th students for each subscale

|  | Grade 5th | Grade 6th | p-value* |
| --- | --- | --- | --- |
|  | Median (Q1 – Q3) | Median (Q1 – Q3) | Bilateral |
| Knowledge (Item 1 a item 3) | 66.67 (41.67 -91.67) | 66.67 (33.33 – 66.67) | 0.223 |
| Communication (Item 4 a item 6) | 58.33 (43.75 – 75.00) | 50.00 (41.67 – 66.67) | 0.232 |
| Attitude  (Item 7 a item 10) | 91.67 (83.33 – 97.92) | 91.67 (83.33 – 100.00) | 0.442 |
| Behaviour  (Item 11 a item 14) | 90.63 (81.25 – 93.75) | 87.50 (73.44 – 95.31) | 0.784 |
| Self-efficacy  (Item 15 a item 17) | 66.67 (50.00 – 83.33) | 66.67 (50.00 – 83.33) | 0.842 |

*The Mann–Whitney U test was used due to the non-normal distribution of the data.
